# Association between Ocular Pseudoexfoliation and Sensorineural Hearing Loss

**DOI:** 10.1155/2014/825936

**Published:** 2014-04-17

**Authors:** Nandini Vijaya Singham, Mimiwati Zahari, Mohammadreza Peyman, Narayanan Prepageran, Visvaraja Subrayan

**Affiliations:** ^1^Department of Ophthalmology, University of Malaya, Lembah Pantai, 59100 Kuala Lumpur, Malaysia; ^2^Department of Otorhinolaryngology, University of Malaya, 59100 Kuala Lumpur, Malaysia

## Abstract

*Background*. Our study aimed to investigate an association between ocular pseudoexfoliation (PXF) and sensorineural hearing loss (SNHL) and to compare them with age and sex matched controls without pseudoexfoliation. *Method*. This was a case-control study of 123 patients which included 68 cases with PXF (at least one eye) and 55 controls without pseudoexfoliation. Pure-tone audiometry (PTA) was done for these patients at sound frequencies taken as important for speech comprehension, that is, 250 Hertz (Hz), 500 Hz, 1000 Hz, and 2000 Hz. *Results*. There were 41 patients with pseudoexfoliation syndrome (PXE) and 27 with pseudoexfoliative glaucoma (PXEG). The majority of patients with hearing loss (60%; *n* = 51) were PXF patients and the remaining 40% (*n* = 34) were controls. Below average hearing thresholds were significantly higher in the pseudoexfoliation group compared to the control group (*P* = 0.01; odds ratio (OR), 3.00; 95% confidence interval (CI), 1.25–7.19). However, there was no significant difference in the mean hearing threshold levels between the three groups (PXE, PXEG, and controls) in either ear (ANOVA, right ear: *P* = 0.46 and left ear *P* = 0.36). *Conclusion*. Our study found an association between PXF and SNHL, confirming that PXF can involve organs in the body other than the eye.

## 1. Background

Pseudoexfoliation (PXF) is an age-related systemic condition characterized by the production and accumulation of abnormal fibrillar extracellular material [1]. Worldwide distribution of this condition varies, being more common in the Scandinavian countries. Forsius H reported a prevalence among Finns of 10% in patients aged 50–69 and higher (25.3%) in the population over age 70 [2].

Ocular pseudoexfoliation is now recognized as a systemic disease as these materials have been found in other parts of the body, namely, the skin, vascular structures, and visceral organs such as the kidney, heart, lungs, and gall bladder as well as in the inner ear [3, 4]. Therefore, PXE may be considered as an ocular manifestation of a systemic disease.

The inner ear is a complex organ. The tectorial and basilar membranes of the inner ear, like the anterior segment structures of the eye, are derived from the neural ectoderm. PXE material has been found on the tectorial and basilar membrane of the inner ear in some studies [5]. Accumulation of pseudoexfoliative material on these structures will interfere in normal hearing threshold levels due to dysfunction of the mechanoreceptors of the ear, resulting in hearing loss.

Normal hearing threshold levels in humans are 0–20 dB, where dB (decibel) is the logarithmic unit of sound pressure which implies loudness of sound energy [6]. A human with normal hearing should have a threshold of 0–20 dB at all frequencies, while in the age standardized. Hearing threshold of 21 dB or more in any one ear is considered as hearing impairment in that ear [6]. Sensorineural hearing loss (SNHL) is the most common form of human sensory deficit and accounts for approximately 70% of cases while encompassing various pathologies in both the inner ear and the auditory nerve [7].

Although all frequencies in the range 250–4000 Hz are relevant for speech comprehension, we have chosen four frequencies that could be important for speech those are 250 Hz, 500 Hz, 1000 Hz, and 2000 Hz [8].

Many studies have been done to find the association of sensorineural hearing loss among pseudoexfoliation patients using speech frequencies of 1000 Hz, 2000 Hz, and 3000 Hz [9]. To find the association between ocular pseudoexfoliation and SHNL, our study used frequencies used from 250 Hz to 2000 Hz.

## 2. Methods and Materials

In this case-control study, all patients who had ocular pseudoexfoliation (syndrome (PXE) or glaucoma (PXEG)) were referred to as the case group and those without pseudoexfoliation as controls. The time frame of the study was from June 2011 to May 2012. All enrolled patients were sent to the otorhinolaryngology clinic for otologic testing and hearing assessment for frequency levels 250 Hz, 500 Hz, 1000 Hz, and 2000 Hz

Inclusion criteria for case group were male and female adults diagnosed with PXE syndrome or glaucoma in either eye, while the control group was patients without ocular pseudoexfoliation matched for age and gender.

Exclusion criteria include patients with any other type of secondary glaucoma other than pseudoexfoliation glaucoma or any ocular or systemic condition having hearing or ear associations. Furthermore patients with history of ear infection, surgery, tympanic membrane perforation, exposure to ototoxic drugs or heavy noise, and upper respiratory tract infection during examination were excluded.

Approval of the ethics committee was obtained from the Ethics Committee of University, Malaya Medical Centre and National Medical Research Registry of Kementerian Kesihatan Malaysia.

A full ophthalmic examination including slit lamp biomicroscopy, gonioscopy (Shaffer), applanation tonometry, pupil dilation, and fundus examination was done. Patients were examined for the presence of PXF (white dandruff-like material) in the pupillary margin on undilated pupils and anterior lens capsule with dilated pupils while the presence of PXF on the trabecular meshwork on gonioscopy was also checked. A Humphrey visual field test for the patients was done. Patients were then sent for otologic examination at the ENT clinic. Hearing assessment (pure tone audiometry) was performed by the audiologist.

*Statistical Analysis.* Tracing of the data was done after completion of the hearing assessment and analyzed using SPSS version 17.00. A significance level of *α* = 0.05 was used for all statistical inferences.

Hence, a *P* value <0.05 will indicate a significant result.

Hearing threshold levels in dB were graded according to severity (normal to profound) and each ear was analyzed separately (Table 1).

## 3. Results

A total of 123 patients were studied, including 68 patients in the ocular pseudoexfoliation group (PXF) and 55 patients in control group. The mean age for the pseudoexfoliation group was 68.5 ± 7.8 years (Mean ± standard deviation) while mean age for the control group was 66.3 ± 7.4. There were 48 male and 75 female patients in the study. Patients with PXF were most commonly found in the age group of 60–69.

Ocular pseudoexfoliation was seen predominantly in females (*n* = 43, 63.23%) in the PXF group. The males had almost equal distribution in both groups. The number of female patients in the PXF group was nearly double the male patients (Pearson, Chi square, *P* = 0.56). The ratio of male to female patients in the PXF group was 1 : 1.7 and in the control group was 1 : 1.4. There was no significant relation found between gender and hearing loss (*P* = 0.1, odds ratio: 0.5, 95% CI (0.2, 1.3)).

The mean IOP was significantly higher in the pseudoexfoliation glaucoma group (right eye: 15.77 mmHg 95% CI (13.87, 17.68); left eye 15.96 mmHg 95% CI (14.03, 17.89)) compared with the control group (*P* = 0.004 for right eye and *P* < 0.001 for the left eye) and left eye in the pseudoexfoliation syndrome group (*P* = 0.07 for the right eye and *P* = 0.004 for the left eye) (ANOVA, Tukey Post Hoc Test). 39.7% (*n* = 27) of patients with pseudoexfoliation had pseudoexfoliative glaucoma. All patients with PXEG were receiving antiglaucoma medication.

A total of 115 of 123 patients (93.5%) in the pseudoexfoliation and control groups were analyzed for sensorineural hearing loss. Six patients had missing data and 2 patients were excluded due to ear pathology. Overall, 73.9% (*n* = 85) had sensorineural hearing loss. Among the 85 patients with hearing loss, 60% (*n* = 51) were pseudoexfoliation patients and the remaining 40% were controls. More than 75% of patients in both groups had bilateral sensorineural hearing loss.

Table 2 shows that the right ear and left ear had a higher prevalence of mild level of hearing loss in the pseudoexfoliation cases as compared with the control group. There were no patients with severe and profound hearing loss in either group.

Patients below average hearing thresholds at speech presentation level were significantly higher in the pseudoexfoliation group compared to the control group (*P* = 0.01; odds ratio (OR), 3.00; 95% confidence interval (CI), 1.25–7.19).

**Figure 1:**
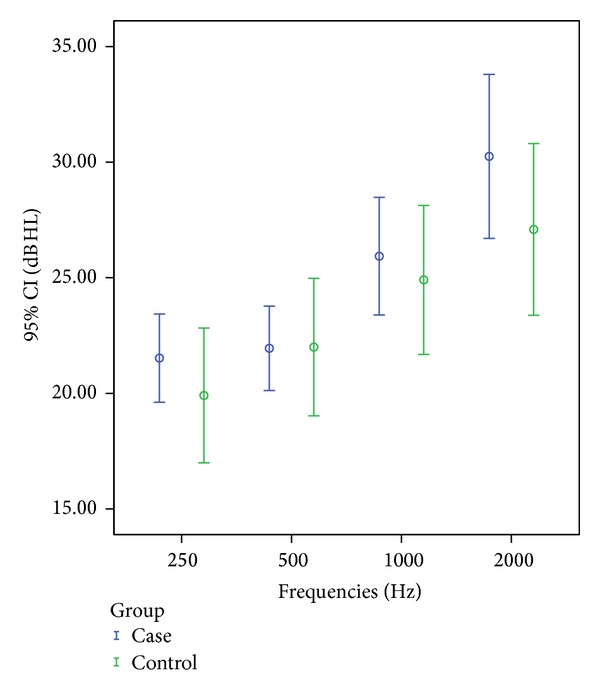
Left ear mean hearing threshold levels (95% confidence interval (CI), decibels (dB), and hearing level (HL) in 4 frequencies in Hz in case and control groups).

In this study frequencies chosen as important for speech level were 250 Hz, 500 Hz, 1000 Hz, and 2000 Hz, tested separately for both ears. Comparisons of means at each frequency in the right ear of the pseudoexfoliation group showed significant higher hearing threshold level at 1000 Hz (*t*-test, *P* = 0.02) when compared with control group, as shown in Figure 1.

**Figure 2:**
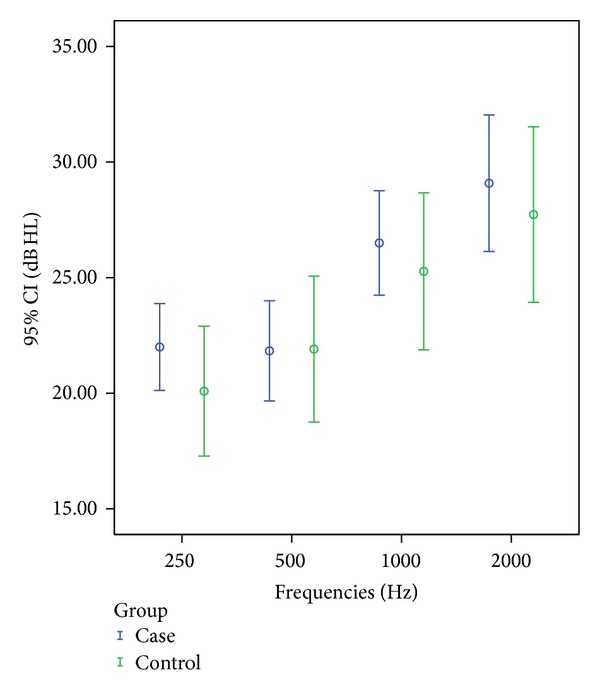
Right ear mean hearing threshold levels (95% confidence interval (CI), decibels (dB), and hearing level (HL) in 4 frequencies in Hz in case and control groups).

Similarly, in the left ear of patients with pseudoexfoliation it was found that mean hearing threshold levels were higher at frequencies 500 Hz (*t*-test, *P* = 0.03) and 1000 Hz (*t*-test, *P* = 0.04) when compared with control group (Figure 2).

## 4. Discussion

Our study investigated the association of SNHL with ocular pseudoexfoliation. Sensorineural hearing loss has many etiologies, but the exact mechanism is still unknown. Based on previous reports pseudoexfoliative material has been identified in the organ of Corti, causing dysfunction in the hearing mechanoreceptors [5, 10]. Deposition of these materials may cause alteration in the vibration induced by sound and can affect the hearing in an individual [10]. A total of 115 patients of 123 patients (93.5%) were analyzed for sensorineural hearing loss and it was found that 73.9% (*n* = 85) had hearing loss. Out of this number, 60% (*n* = 51) were pseudoexfoliation patients and the remaining 40% (*n* = 34) were controls. A study done by Yazdani et al. found that SNHL in pseudoexfoliation syndrome (PXE) patients was more common (88.4%) than in controls (53.6%) [9].

In our study, bilateral sensorineural hearing loss was more prevalent among the pseudoexfoliation group. According to the severity of the hearing loss, most of the patients had mild SNHL in both groups. There was no significant difference between the laterality of the hearing loss. This supports the findings of Cahill et al., who found no significant relationship between laterality of sensorineural hearing loss in either ear in patients with ocular pseudoexfoliation [11].

Comparison of the mean value of selected speech frequencies between pseudoexfoliative cases and controls showed that there was a significant relationship of higher threshold levels at frequency 1000 Hz in the right ear of the pseudoexfoliation group. Similarly, it was significant in the left ear for frequencies 500 Hz and 1000 Hz. Ozkan et al. found that there was a significantly higher prevalence of hearing threshold levels at 500 Hz, 1000 Hz, and 2000 Hz in their population of pseudoexfoliative cases when compared with controls [12]. Furthermore, impaired acoustic function in patients with pseudoexfoliation was demonstrated by Detorakis et al. [13].

There were a few limitations in the study. The case-control design of the study is one of the limitations in the current project, as is the lack of nomogram for normal hearing in our population for comparison of hearing threshold levels with the ISO 7029 standard, which is based on data from Caucasian populations [14]. Also the restriction of the study to the frequencies 250–2000 Hz is a possible limitation. Also the audiometric testing used here is pure tone audiometry which has the advantage of being rather sensitive to any form of hearing impairment and, however, delivers only limited information on the mechanisms involved in hearing loss of a given patient.

By demonstrating a higher prevalence of sensorineural hearing loss in ocular pseudoexfoliation patients we support other studies that suggest it may represent a manifestation of a systemic disease in multiple organs and tissues throughout the body.

Ocular pseudoexfoliation is now recognized as a systemic disease as these materials have been found in other parts of the body, namely, the skin, vascular structures, and visceral organs such as the kidney, heart, lungs, gall bladder, and the inner ear. Therefore, PXE may be considered as an ocular manifestation of a systemic disease.

## 5. Conclusion

Our study found an association between ocular pseudoexfoliation and sensorineural hearing loss. This study could generate interest in further research in the field of systemic associations of pseudoexfoliation.

## Figures and Tables

**Table 1:** Hearing threshold was used in our study.

Hearing threshold grading	dB HL
Normal	0–20
Mild	21–40
Moderate	41–60
Moderate-severe	61–80
Severe	81–100
Profound	>100

**Table 2:** Hearing loss graded according to severity in both ears.

Ear	Hearing loss grading (dB)	Group		Total
		PXF	Control	
Right	Normal (0–20)	12	24	36
	Mild (21–40)	37	20	57
	Moderate (41–60)	11	10	21
	Moderate-severe (61–80)	0	1	1
	Severe (81–100)	0	0	0
	Profound (>100)	0	0	0
Total		**60**	**55**	**115**

Left	Normal	16	25	41
	Mild	32	19	51
	Moderate	9	10	19
	Moderate-severe	2	1	3
	Severe	0	0	0
	Profound	0	0	0
Total		**59**	**55**	**114**
